# Scrub Typhus among Febrile Patients Admitted to the Department of Medicine in a Tertiary Care Centre: A Descriptive Cross-sectional Study

**DOI:** 10.31729/jnma.8208

**Published:** 2023-07-30

**Authors:** Sanjeeb Shrestha, Shanti Pradhan, Niranjan Shrestha, Mitesh Karn

**Affiliations:** 1 Department of Biochemistry, Gandaki Medical College Teaching Hospital, Pokhara, Kaski, Nepal; 2 Department of Public Health, School of Health and Allied Sciences, Pokhara University, Pokhara, Kaski, Nepal

**Keywords:** *acute febrile illness*, *complications*, *scrub typhus*

## Abstract

**Introduction:**

Scrub typhus is an acute febrile illness caused by obligate intracellular bacteria of the family Rickettsia after an incubation period of 6-21 days. The disease may lead to several complications such as acute hepatitis, sepsis, myocarditis, respiratory distress and disseminated vasculitis if not treated properly. The aim of the study was to find out the prevalence of scrub typhus among febrile patients admitted to the Department of Medicine in a tertiary care centre.

**Method:**

This descriptive cross-sectional study was conducted among febrile patients admitted to the Department of Medicine in a tertiary care centre from 12 November 2020 to 11 May 2023 after obtaining ethical approval from the Institutional Review Committee (Reference number: 26/076-077). All patients with acute febrile illness from 17 years to 70 years were included. Patients with an acute febrile illness diagnosed with other infections such as dengue, leptospirosis, typhoid, brucellosis, malaria, and COVID-19 were excluded. Convenience sampling was done. Point estimate and 95% Confidence Interval were calculated.

**Results:**

Among 640 patients presenting with acute febrile illness, 38 (5.94%) (4.11-7.77, 95% Confidence Interval) patients were diagnosed to have scrub typhus. Among 38 patients, 2 (5.26%) patients suffered from acute hepatitis, and 1 (2.63%) each suffered from sepsis, myocarditis, disseminated vasculitis and respiratory distress.

**Conclusions:**

The prevalence of scrub typhus among patients admitted to the Department of Medicine was found to be slightly higher than in other studies done in similar settings.

## INTRODUCTION

Scrub typhus is an acute infectious disease caused by *Orientia tsutsugamushi* transmitted to humans by the bite of larval trombiculid mite (chiggers), an arthropod vector. A sudden upsurge of cases has been reported throughout the country for three years after the 2015 earthquake in Nepal and this underlines the risk of epidemic outbreak. It causes generalized vasculitis that can involve multiple organs. Reported complications include hepatitis, acute renal failure, sepsis, myocarditis, meningoencephalitis, disseminated intravascular coagulation (DIC), and multi-organ-failure.^1,2^

This disease is a hazard in many parts of South East Asia and beyond. It is the first rickettsial infection to show antibiotic resistance. Many patients may die from complications following the disease. So, close attention should be paid to the diagnosis and the disease severity.^3-4^

Hence, the aim of the study was to find out the prevalence of scrub typhus among febrile patients admitted to the Department of Medicine in a tertiary care centre.

## METHODS

This descriptive cross-sectional study was conducted among febrile patients admitted to the Department of Medicine in Gandaki Medical College Teaching Hospital (GMCTH), Pokhara, Kaski, Nepal after obtaining ethical approval from the Institutional Review Committee (Reference number: 26/076/077). Data from 12 November 2020 to 11 May 2023 was collected. Written informed consent was obtained from the study participants. All patients with acute febrile illness from 17 years to 70 years were included. Patients with an acute febrile illness diagnosed with other infections such as dengue, leptospirosis, typhoid, brucellosis, malaria, and COVID-19 were excluded. Convenience sampling was used. The sample size was calculated using the formula:


$$\begin{array}{ll} \text{n} & ={\text{Z}}^{2}\times \frac{\text{p}\times \text{q}}{{\text{e}}^{\text{2}}}\\ & =1.96^{2}\times \frac{0.137\times 0.863}{0.03^{2}}\\ & =505 \end{array}$$

Where,

n = minimum required sample size
Z = 1.96 at 95% Confidence Interval (CI)
p = prevalence of scrub typhus taken from a previous study, 13.7%^5^
q = 1-p
e = margin of error, 3%

The calculated sample size was 505. However, 640 patients were included in the study.

After taking written informed consent, the demographic data, clinical features, past and present history of illnesses, and vital signs were collected in a preformed questionnaire. About 10 ml venous blood was collected by trained laboratory staff, aseptically with the standard operating procedure, and divided into three aliquots for serological, biochemical, and haematological tests in becton dickinson vacutainer, yellow colour-coded and lavender colour-coded tubes. The collection and transportation of specimens were done according to the manufacturer's instructions. Blood samples collected were received in the central laboratory. Scrub typhus was diagnosed with IgM ELISA Kit by detecting IgM antibodies in the serum sample.^6^ Hematologic test was done by automated haematology analyzer, 5 parts, HEMA-D6051 using a flow-cytometer principal according to the manufacturer's instruction.

Data were collected and entered in Microsoft Excel 2007 and were analysed in IBM SPSS Statistics version 11.5. Point estimate and 95% CI were calculated.

## RESULTS

Among 640 patients with acute febrile illness, scrub typhus was seen in 38 (5.94%) (4.11-7.77, 95% CI) patients. Out of 38 scrub typhus patients, 26 (68.42%) were female and 12 (31.58%) were male. Thirteen (39.47%) patients were treated on an outpatient department (OPD) basis and 25 (65.78%) patients were admitted to the hospital (Table 1).

**Table 1:** Duration of hospital stay (n = 38).

Duration of hospital stay	n (%)
2-5 days	18 (47.36)
>5 days	7 (18.42)

Among 38 patients, the resolution was seen in 31(81.58%) (Table 2).

**Table 2:** Complications of scrub typhus (n= 38).

Complications	n (%)
Acute hepatitis	2 (5.26)
Sepsis	1 (2.63)
Congestive heart failure	1 (2.63)
Mortality following ARDS	1 (2.63)
Encephalitis leading to hemiparesis	1 (2.63)
DIC	1 (2.63)
Resolution	31 (81.58)

## DISCUSSION

Among 640 patients with acute febrile illness, scrub typhus was seen in 38 (5.94%) patients. This finding was lower than the finding in a similar study.^5^

The mortality rate in our study was 1 (2.63%). The rate is lower than that of a nationwide study, where 101 cases were reported from 16 districts and eight cases succumbed due to illness, accounting for an 8% mortality rate.^3^ This could be attributed to increased awareness of the non-specific presentation of scrub typhus, early diagnosis and treatment in our tertiary care centre.The disease was more prevalent in females 26 (68.42%) as compared to males. In a similar study, it was found that there was no significant difference in the gender-wise prevalence.^5^

The disease in our study invariably presented as an acute febrile illness with non-specific signs and symptoms. The similarity in clinical presentations and diverse causative agents complicate the diagnosis of the disease. The cases were categorized into non-severe, severe and deceased groups. In our study, 25 (34.21%) non-severe patients had milder symptoms. They got treatment with doxycycline and/or azithromycin in the OPD and did not require admission. This syndromic approach is a simple and inexpensive treatment protocol which covers other infectious diseases as well. This might be the reason for the fewer complications and death in our setting. This finding is similar to a study done in Dhulikhel Hospital, Nepal.^7^ Severe scrub typhus showed involvement in one or the other systems like acute hepatitis, sepsis, cardiac failure, ARDS, encephalitis, DIC and death. These complications were encountered in 7 (18.42%) cases. This data concurs with the study done in Telangana, south India.^5^ In our study, the most common complication was acute hepatitis 2 (5.26%). The findings were similar to the study done in South-Korea.^8^

Likewise, acute respiratory distress syndrome (ARDS) is a serious complication of scrub typhus and delay in the use of appropriate antibiotics can lead to mortality. In our study, 1 (2.63%) of cases suffered from (ARDS). The major cause of mortality was delay in diagnosis. A study done in the southwestern part of China reported ARDS as a complication of scrub typhus in 11.1% of cases, a quarter of which suffered from mortality.^9^ Hence, it is important to suspect and look for respiratory symptoms to establish the diagnosis early and prevent morbidity as well as mortality. Similarly, cardiac failure was reported in 1 (2.63%) of the patients as a consequence of myocarditis. These findings were similar to the studies conducted in Taiwan.^10^

Another interesting observation was the involvement of the central nervous system in the studied patient. Encephalitis is less defined in the literature and was observed in 1 (2.65%) patients in our study. The findings were consistent with the study done in Korea.^11^ Laboratory findings were similar to that reported by Thai children.^12^ Furthermore, 1 (2.63%) patient had an abnormal coagulation profile and there was oozing from the venipuncture site, petechiae, and ecchymoses leading to the development of DIC. These findings were in accordance with the case reports from Japan.^13^

Our study is a single-centered and the sample size is also small. Therefore, the results cannot be generalized in a larger population.

## CONCLUSIONS

The prevalence of scrub typhus among patients admitted to the Department of Medicine was slightly higher than in other studies done in similar settings. Hence, scrub typhus should be considered as a differential diagnosis in febrile cases because the clinical spectrum often overlaps and diagnosis becomes difficult.
